# Complementary Phenotyping of Maize Root System Architecture by Root Pulling Force and X-Ray Imaging

**DOI:** 10.34133/2021/9859254

**Published:** 2021-11-10

**Authors:** M. R. Shao, N. Jiang, M. Li, A. Howard, K. Lehner, J. L. Mullen, S. L. Gunn, J. K. McKay, C. N. Topp

**Affiliations:** ^1^Donald Danforth Plant Science Center, Saint Louis, MO, USA; ^2^Department of Agricultural Biology, Colorado State University, Fort Collins, CO, USA

## Abstract

The root system is critical for the survival of nearly all land plants and a key target for improving abiotic stress tolerance, nutrient accumulation, and yield in crop species. Although many methods of root phenotyping exist, within field studies, one of the most popular methods is the extraction and measurement of the upper portion of the root system, known as the root crown, followed by trait quantification based on manual measurements or 2D imaging. However, 2D techniques are inherently limited by the information available from single points of view. Here, we used X-ray computed tomography to generate highly accurate 3D models of maize root crowns and created computational pipelines capable of measuring 71 features from each sample. This approach improves estimates of the genetic contribution to root system architecture and is refined enough to detect various changes in global root system architecture over developmental time as well as more subtle changes in root distributions as a result of environmental differences. We demonstrate that root pulling force, a high-throughput method of root extraction that provides an estimate of root mass, is associated with multiple 3D traits from our pipeline. Our combined methodology can therefore be used to calibrate and interpret root pulling force measurements across a range of experimental contexts or scaled up as a stand-alone approach in large genetic studies of root system architecture.

## 1. Introduction

In maize, the entirety of primary, seminal, lateral, crown, and brace roots together form a complex architecture which controls the plant's ability to effectively acquire water, scavenge nutrients, and resist lodging [1]. As a result, root growth and development are fundamental to overall plant development and competitiveness [2], and several prominent large-effect, loss-of-function mutants in cereal seedling root development have been identified and reviewed previously [3, 4]. However, root system architecture of mature, field-grown plants at the quantitative level has been understudied and underutilized due to the relative difficulty in obtaining measurements, with significant tradeoffs intrinsic to any particular phenotyping method [5, 6]. Nevertheless, because root growth is highly plastic and affected by environmental conditions such as substrate moisture and texture [7, 8], field-based studies are valuable despite their challenges.

In its simplest form, root phenotyping of crop species such as maize or rice can be performed by manual measurement of a limited set of amenable traits, such as root mass, length, width, or the growing angle, either in soil or soil-free conditions. Currently known genes controlling quantitative root system architecture traits in rice were identified using such measurements, including *PSTOL1* [9], *DRO1* [10], and a recent *DRO1* homolog [11]. In field conditions, additional techniques for quantifying roots exist, such as the use of minirhizotrons, soil core sampling, and measuring of root pulling force [12–15]. Historically, root pulling force (RPF) has been useful as a field assay because of its simplicity; it also has the greatest potential for large-scale phenotyping and has been applied to both monocots and dicots [16–21]. While RPF is generally correlated with greater root biomass and branching, more nuanced interpretations and its association with recently tractable architectural measurements have yet to be established, and the relationship between RPF above-ground traits is even more complex due to pleiotropy and other effects. In a *B. napus* doubled haploid mapping population, RPF was positively associated with later flowering time and subsequently lower grain yield [22], while in maize, QTLs for RPF colocalized with leaf abscisic acid concentration [23], suggesting that additional context-specific measurements are required to interpret RPF.

More intricate phenotyping of root system architecture can be performed upon two-dimensional images of either field-excavated root crowns, or young gel-media grown root systems, followed by analysis with specialized software [24–31]. Such methods have been used to quantify root system architecture in diverse crops such as maize, wheat, rice, and cowpea [32–35]. However, 2D-based measurements have a limitation in that images are typically taken from only one or two camera perspectives, with information lost from roots occluding each other in the image.

As a result, interest and capacity towards three-dimensional root phenotyping have been increasing, driven in part by technical advances and interdisciplinary approaches [36]. For example, young cereal plants grown in a gel-based media can be imaged over a 360° rotation, allowing digital reconstruction in 3D and high-throughput feature extraction [37, 38]. By scaling this technique to mapping populations, studies have identified new univariate or multivariate root QTLs, demonstrating the value of high-throughput and high-information-content trait capture for dissection of plant architecture [39, 40]. Other 3D-based solutions include the use of X-ray computed tomography (XRT), which is capable of imaging any plant structure, including roots within soil based upon physical density properties [41–48]. While XRT has been applied to plant physiology in some form for nearly two decades, instrument accessibility and technical limitations typically restrict its use to small plant structures, low throughput, and/or limited fields of view.

Here, we integrate two protocols, first sampling via RPF and washing mature, lignified, field-grown maize root crowns, followed by imaging via XRT and trait quantification for over 290 roots across multiple field seasons. By imaging the roots absent of soil or other media, scanning and segmentation times were significantly reduced such that replication across two environments and/or two time points was possible. We extracted up to 71 3D features for each root crown sample, including up to 65 traits with significant variation between genotypes, as well as root shape or distributional traits, which showed differences between experimental contexts. The median broad-sense heritability across all traits ranged from 0.23 to 0.56, depending on the germplasm and conditions. Finally, we examined covariance between 3D traits and RPF values to identify correlations between high-resolution phenomics and high-throughput field data. This study therefore demonstrates how XRT can provide insights into the root architectural attributes that influence RPF, which can then be used together for more informative studies in the mapping and breeding of root traits, including in multienvironment analyses.

## 2. Materials and Methods

### 2.1. Experimental Design

All plants were grown at the Colorado State University Agricultural Research Development and Education Center in Fort Collins, CO, USA (40.649 N, -105.000 W), in 2017 and 2018. 260 genotypes from the *Genomes 2 Fields* (G2F) germplasm (https://www.genomes2fields.org/) were planted in May 2017 in a split-plot design with full irrigation or limited irrigation (drought) treatments, with two field replicates per treatment for a total of 1060 plots. Prior to planting, the field was fertilized with nitrogen at 65 lbs per acre.

The Shoot Apical Meristem (SAM) diversity panel [49] along with 11 hybrid and 4 inbred check lines, for a total of 390 genotypes, was planted in May 2018 using a split-plot design with full irrigation or limited irrigation (drought) treatments, with three field replicates per treatment. Prior to planting, the field was fertilized with nitrogen at 190 lb per acre. Root systems were harvested at 9 weeks after planting (time point 1) and again at 16 weeks after planting (time point 2).

In both the G2F and SAM experiments, each plot consisted of two 12-foot rows with 30-inch spacing between rows and 9-inch spacing between plants within rows, corresponding to a planting density of 62000 plants/ha. The irrigated treatments received approximately 1 inch of water per week, while the drought treatments were irrigated until well established (approx. 5 weeks after planting) and then received only natural precipitation (103.8 mm and 69.9 mm in the 2017 and 2018 growing seasons, respectively), except at the root harvesting when it also received irrigation to homogenize the root harvesting process.

### 2.2. Field Phenotyping and 2D Root Imaging

**Figure 1:**
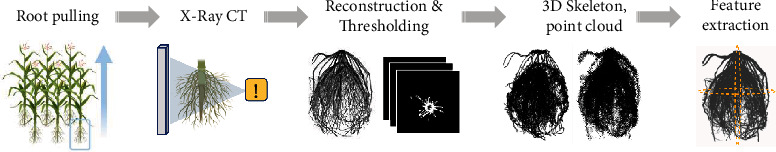
Pipeline for 3D root imaging using X-ray computed tomography. Samples are excised from the soil in the field using the root pulling force method, and the measurement is recorded. The root crown is washed, dried, and then imaged using the NorthStar Imaging X5000 (see Methods) to generate radiographs as the sample is rotated 360° across the vertical axis. From the radiographs, a 3D reconstruction is generated using the FDK algorithm. Slices along the vertical axis are exported for automated thresholding, from which a skeleton and point cloud model of the root crown are generated. 3D root traits are then measured from the skeleton and point cloud and analyzed.

The protocol used for root pulling and harvesting was similar to that in [22]. Briefly, all plants were irrigated 24 hours prior to sampling to homogenize soil conditions at root harvest. Maize plants were tied at the base of the stem, just above the root crown, with a rope attached to a dynamometer. The root system was extracted from the soil by vertical manual pulling (Figure 1), with the force (Kg) needed for extraction measured using a hand-held Imada DS2 digital force gauge (Imada Inc., Northbrook, IL, USA). Within each field treatment (full vs. limited irrigation), two roots per genotype were harvested from the G2F population and an average of 4 roots per genotype (across two time points) were harvested for the SAM population. Based on root pulling force values, 30 representative genotypes from the G2F 2017 experiment and 20 representative genotypes from the SAM 2018 experiment (Table S1, Figure S1A) were selected for further analysis. After pulling, root samples were washed to remove all remaining soil, allowed to air dry, and weighted for dry mass (g) before imaging.

Roots from the G2F 2017 experiment were also imaged in 2D (Figure S1F) using a photography station equipped with a Sony a7 II mirrorless camera and Sony FE 28 mm f/2.0 lens, mounted 31 inches above the stage. Roots were placed horizontally on a flat surface with a black cloth background, with lighting provided by a LS Photography PPH67 ring light, and the resulting images (60 pixels/cm) were then cropped and analyzed using DIRT [25] on CyVerse with threshold = 10, scale marker = 39, “require segmentation” checked, and “require stem reconstruction” unchecked.

### 2.3. 3D Root Imaging and Feature Extraction

For 3D phenotyping of samples from the selected G2F and SAM populations (*N* = 107 and 187, respectively), root crowns were clamped at the stem with a small vise and imaged using a North Star X5000 X-ray system (North Star Imaging, MN, USA) (Figure 1). Depending on the physical dimensions of the largest root crown sample within a given sample batch, the object stage and detector were positioned 1036–1055 mm and 1186–1210 mm away from the object source, respectively. After gain calibration, the X-ray source was set to 70 kV and 1500–1700 *μ*A and a focal spot size of 105–119 microns. Each root crown sample was continuously imaged within a single 360° rotation using efX-DR (North Star Imaging), generating 1800 radiographs per sample at 10 fps (100 ms integration time). To provide an internal calibration of the image geometry, a fixed standard (15 mm large tool, North Star Imaging) was imaged with each sample batch. The radiographs were then reconstructed using efX-CT (North Star Imaging) and exported as an unadjusted RAW volume, resulting in a voxel size of 109–113 *μ*m depending on the sample batch.

For each sample, the RAW volume was converted to 2D slices using the custom Python script *raw2img*. The slices were then thresholded, binarized, and skeletonized using the custom scripts *batch-segmentation*, which performs basic thresholding of the roots from air in the 3D volume, and *batch-skeleton*, which calls the skeletonization and feature extraction pipeline previously developed and described in [31, 39], and [50]. These 19 traits were computed, and where applicable, converted to physical units by dividing values by the voxel size, the voxel size squared, or the voxel size cubed. In addition, 52 root traits were newly added to the pipeline for this study, which predominantly focus on 3D distribution of roots in the root crown. Mean, standard deviation, skewness, kurtosis, energy, entropy, and smoothness from the distributions of root volume (estimating root mass), convex hull, and solidity were calculated using the method described in [51]. Fractal dimension, which measures the degree to which root subsections approximate a smaller copy of the whole root crown [52], was estimated by taking the 2D projection of the 3D volume, then calculated using a similar approach to that described in [53]. DensityS features are computationally similar to plant compactness traits described in [54].

A list and basic description of root features measured using *batch-skeleton* are available in Table S2. The raw phenotype data is available in Data File S1, with an example of a root crown 3D reconstruction and models shown in Figure S1B-F. A more extensive description of trait implementations, all scripts used for image processing and feature extraction, and links to repositories required to reproduce the work are available at https://github.com/Topp-Roots-Lab/3d-root-crown-analysis-pipeline/

### 2.4. Statistical Analysis

All downstream (i.e., post feature extraction) analysis was performed in the R statistical computing environment. Initially, principal component analysis using all 71 3D root traits was used to identify large outliers, leading to the removal of 2 samples in the G2F 2017 data and 3 samples in the SAM 2018 data. Additionally, for all univariate analyses, outliers within each trait were identified and omitted if they were beyond the 1st quartile minus 1.5 ∗ interquartile-range or the 3rd quartile plus 1.5 ∗ interquartile-range.

After univariate outlier removal, analysis of variance (ANOVA) was performed for each trait using the *car* package [55]. Subsequently, the ANOVA *p* values from *car* were adjusted using the Benjamini-Hochberg method to account for multiple testing and control the false discovery rate to an adjusted *p* value < 0.05. Individual two-sample comparisons as seen in boxplots were performed using Mann-Whitney *U* tests. Correlations between root traits were calculated using Spearman's correlation coefficient. Linear regressions were performed using the *lm* function in R.

Variance components were estimated by using the *lme4* package [56] to fit the linear model *Y*_*ijk*_ ~ *G*_*i*_ + *E*_*j*_ + (*G*∗*E*)_*ij*_ + *e*_*ijk*_, where *Y* is the phenotypic value, *G*_*i*_ is the *i*^th^ genotype, *E*_*j*_ is the *j*^th^ environment, (*G*∗*E*)_*ij*_ is the interaction between the *i*^th^ genotype and the *j*^th^ environment, and *e*_*ijk*_ is the residual error of the *k*^th^ sample from the *i*^th^ genotype and *j*^th^ environment. Broad-sense heritability was calculated using the equation *H*^2^ = *σ*_*G*_/(*σ*_*G*_ + *σ*_*GxE*_/*e* + *σ*_residual_/*re*) where *σ*_*G*_ is the estimated phenotypic variance due to genotype, *σ*_*GxE*_ is the estimated phenotypic variance due to genotype *x* environment, *σ*_residual_ is the residual variance, *e* is the number of environments, and *re* is the average number of biological replicates per genotype across both environments [57]. This heritability estimator is optimized as a predictor of the response to selection. For the SAM 2018 data, variance components and broad-sense heritability were calculated separately for the two time points.

Principal component analysis (PCA) of the 3D root data within the G2M and SAM experiments was performed using the base R *prcomp* function. For PCA-LDA, PCA was performed upon each genotype subset, and the number of principal components required to explain 90% of the trait variance was used as inputs into the LDA function from the *MASS* package [58]. The *randomForest* [59] and *caret* [60] R packages were used for random forest classification, with *mtry* and *ntree* parameters found using a grid search approach between every combination of *mtry* between 1 and 20 and *ntree* values of 500, 1000, 2500, and 5000. Parameters giving the best accuracy were kept, as calculated by 10-fold cross-validation repeated 3 times. From the final random forest models, the proximity matrix was calculated and nonmetric multidimensional scaling was used to visualize the distances between samples.

## 3. Results

### 3.1. Field and 3D Phenotyping Capture Variation in Maize Root System Architecture

In each of two field seasons, maize genotypes (30 from the G2F panel and 20 from the SAM panel) were grown under two different irrigation treatments, providing two environments in terms of soil moisture. At the designated time point(s) for sampling (see Methods), root crowns were excavated by root pulling force. The root crowns, which maintain their overall 3D structure due to lignification, were washed clean and subsequently imaged using a Northstar X5000 X-ray computed tomography system (Figure 1). In total, 71 traits from the 3D volumes were extracted and used for analysis (Table S2). Correlations between the most directly comparable 3D traits and 2D traits (via DIRT) were as we expected—for example, 3D surface area and 3D volume had a Pearson correlation coefficient of 0.754 and 0.703 to 2D area, respectively (Figure S2).

To assess the degree to which traits derived from field-pulled root crown samples would respond to selection, we estimated broad-sense heritability (*H*^2^) in the G2F experiment for each 3D and 2D trait, as well as for RPF (Figure S3). Traits related to overall root crown size showed similar *H*^2^ values between 3D measurements (e.g., 3D surface area *H*^2^ = 0.47) and 2D measurements (e.g., 2D area *H*^2^ = 0.44). The traits with the highest heritability, however, were 3D-derived maximum root count (*H*^2^ = 0.76) and average root radius (*H*^2^ = 0.74), illustrating where 3D root phenotyping is particularly adept. Among 2D traits, high heritability did not necessarily result in high association with RPF, although some traits such as 2D area had a strong positive association (Figure S3E-F). Nevertheless, depending on the experimental conditions and amount of replication, it is probable that many root traits—though computationally extractable—have questionable value due to high background noise and sensitivity to sampling variation. In total, for example, 19 3D traits and 33 2D traits had calculated *H*^2^ values of less than 0.05; therefore, both 3D and 2D root traits must be screened and evaluated for a given data set before drawing conclusions. RPF itself had a *H*^2^ value of 0.67 in the G2F experiment, which is high for a physical field-based root assay, and competitive with some of the architectural traits measured from 3D images.

In the SAM experiment, broad-sense heritability for 3D traits (2D traits were not captured here) was calculated separately within each time point (Figure S4A-C). In general, *H*^2^ values here were higher than in the G2F experiment, which reflects a combination of the field conditions, genetic variation, and sample sizes. As an average across both time points, root crown width (“HorEqDiameter,” mean *H*^2^ = 0.81), fractal dimension top view (mean *H*^2^ = 0.81), maximum root count (mean *H*^2^ = 0.80), convex hull volume (mean *H*^2^ = 0.79), and surface area (mean *H*^2^ = 0.79) were among the most heritable traits, although the individual performance of these traits fluctuated depending on the time point. However, the average heritability across all 3D traits was only slightly higher at first time point (0.510) than at second time point (0.505), suggesting that heritability of most root traits is relatively static over this time span. One interesting exception to this is RPF itself, which had a *H*^2^ value of only 0.59 at the first time point, but significantly increased to a *H*^2^ value of 0.85 by the second time point. This indicates that RPF measurements taken later in the plant life cycle may be more informative and reliable for the purposes of distinguishing genotypic differences in maize root system architecture, as well as for breeding. Overall, however, traits with higher average heritability across time points tended to also have a greater correlation in measurements between time points (Figure S4D).

**Figure 2:**
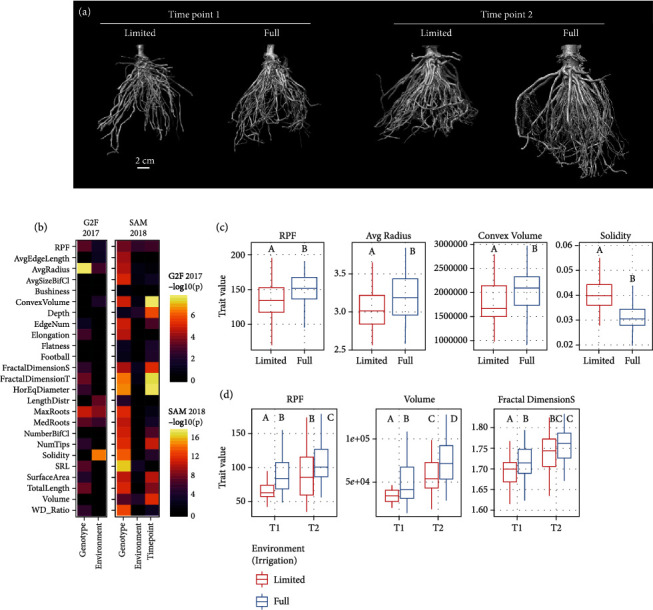
RPF and 3D global root system architecture traits are affected by genotype, environment, and developmental time point. (a) 3D reconstructions from X-ray imaging of genotype Tx601 root crowns in the SAM 2018 experiment, at the two time points (9 vs. 16 weeks) and from each environment (limited vs. full irrigation). (b) ANOVA for genotype, environment, and time point (in SAM 2018) effects upon RPF and 3D root traits (adjusted *p* < 0.05); for legibility, G2F 2017 and SAM 2018 experiments were separately scaled, and nonsignificant features were set at a -log10(*p*) value of 0. (c) Boxplot of selected traits significantly different between the two environmental conditions in the G2F 2017 experiment (Mann-Whitney *U* test *p* < 0.05). (d) Boxplot of selected traits significantly different between the two development time points and/or the two environmental conditions in the SAM 2018 experiment (Mann-Whitney *U* test *p* < 0.05).

Focusing on 3D root traits and RPF, we subsequently wanted to examine whether genotype and environment effects were significant factors on a trait-by-trait basis (Figure 2). Using analysis of variance (ANOVA), in the G2F experiment, we detected 21 root traits where genotype had a significant effect and 35 traits where the environment (irrigation regime) had a significant effect (Figure 2(b), Figure S5A, Figure S6). Root traits affected by both genotype and environment include RPF, average root radius, median/maximum number of roots, convex hull skewness, and solidity in several regions along the middle of the root crown. Nonparametric tests for differences between environments confirmed that RPF, average root radius, and convex hull volume, for example, were higher in the high irrigation environment, whereas solidity was higher in the low irrigation environment (Figure 2(c)). These meet expectations of soil moisture effects on root system architecture (e.g., more expansive growth under higher moisture availability), providing confidence to our 3D phenotyping.

In the SAM experiment, the situation was somewhat reversed: in support of the overall higher trait heritability, a remarkable 65 root traits had a significant effect from genotype, but only 17 traits had a significant effect from environment, while 37 traits had a significant effect from time point (Figure 2(b), Figure S5A, Figure S7). Traits such as RPF, surface area, volume, root crown depth, fractal dimension side view, and biomass distribution skewness were affected by all three variables. Again, nonparametric tests for differences in RPF, volume, and fractal dimension side, for example, confirmed the impacts of environment and time point as detected by ANOVA (Figure 2(d)). The somewhat divergent trends in 3D root phenotypes between the G2F and SAM experiments, however, indicate that additional generalizations about root system architecture and how growth plasticity relates to it may be difficult to come by, as root variation is highly dependent on the experimental conditions, population, and developmental stage, similar to other quantitative traits [61]. Indeed, although the sample sizes here precluded strong statistical power to test genotype-environment interactions using ANOVA, variance component analysis suggests that such interactions may have a significant influence on a number of root architecture traits (Figure S3B, Figure S4B-C).

### 3.2. Root Architecture Relationships and Correspondence to Root Pulling Force

**Figure 3:**
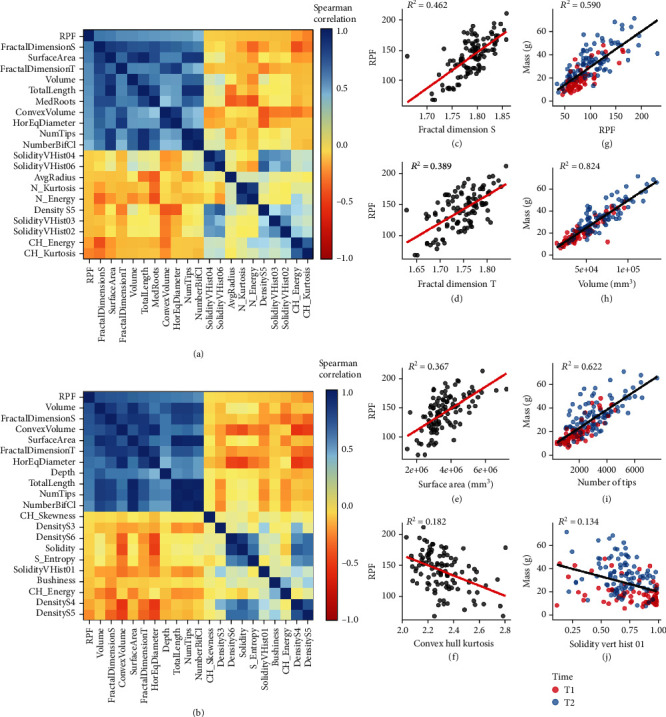
RPF and 3D root system architecture traits are strongly associated with each other and root mass (grams). Heatmap of RPF and its 20 most correlated traits (10 most positive and 10 most negative) in the G2F 2017 (a) and SAM 2018 (b) datasets. Regression of example traits positively correlated to RPF such as fractal dimension side/top (c, d) and surface area (e), and example traits negatively correlated to RPF such as convex hull volume (f), within the G2F 2017 dataset. Regression of example traits positively correlated to root mass (grams) such as RPF (g), volume (h), and number of tips (i), and example traits negatively correlated to root mass (grams) such as solidity vertical histogram-01 (j), within the SAM 2018 dataset. Adjusted *R*-squared values for c–j shown in respective plot insets.

Root pulling force has been used historically and recently as a proxy for root mass and root volume. Nevertheless, to have additional and more detailed information on the architectural changes that RPF measures would increase its utility as a field assay. We first calculated correlations between RPF and 3D root phenotype across all measured samples, irrespective of genotype and environment, or time point in the case of the SAM data. In both experiments, RPF was most correlated with root volume, fractal dimension, surface area, total root length, root crown width, number of bifurcating clusters, and number of root tips. (Figures 3(a) and 3(b), Table S3). Traits negatively correlated with RPF were generally weaker and less consistent between the two experiments but did include convex hull energy (a measure of root system uniformity) and Density S5 in both cases.

Associations between root pulling force and root system architecture traits are most useful if they are not only significantly correlated but also exhibit a close linear relationship. Regression analysis between RPF and positively correlated 3D architecture traits (as observed in the G2F experiment), such as fractal dimension and surface area, showed a reasonably good fit (Figures 3(c)–3(e)). In contrast, there was a relatively poor fit with convex hull kurtosis, the most negatively correlated trait (Figure 3(f)). These may in part be due to sampling error or noise, but also because multiple root characteristics that may not be strongly correlated to each other nevertheless each contribute to RPF in various ways. Nevertheless, the regression fit between physical root mass (i.e., root crown weight) in the SAM experiment and RPF or other positively correlated 3D architecture traits was extremely high, while again relatively poor with negatively correlated traits such as solidity in the upper root crown (Figures 3(g)–3(j)). This high goodness-of-fit was not a by-product of regression between two time points; rather, regression between physical root mass (g) and these 3D traits remained high even when observing trends and regressions within each time point (Figure S5B-J). Furthermore, regression fit was typically higher in time point 1 than in time point 2, which might be due to root crown traits beginning to diverge in ways more independent of root mass, such as in architectural and spatial orientation, which could nonetheless contribute to RPF.

To explore the degree to which trends across multiple traits may be associated with RPF, we performed principal component analysis (PCA) from the G2F and SAM data using the 3D-based root phenotypes alone (Figure S8A, E). In the G2F data, RPF was more tightly associated with principal component 2 (Figure S8B), which was primarily composed of traits related to overall size, e.g., surface area, volume, total root length, and number of root tips, but also significantly composed of 3D biomass distribution traits (Figure S8I). On the other hand, in the SAM data, RPF was more tightly associated with principal component 1 (Figure S8F), which as with the G2F data was primarily composed of traits related to overall size, including surface area, volume, and total root length, and additionally fractional dimension side/top, but notably not of 3D biomass distribution traits (Figure S8J). Both PC1 and PC2 were statistically different between the two environmental conditions in the G2F data and between the two time points in the SAM data (Figure S8C-H), but the differences between the G2F and SAM results here likely derive from the fact that much of the phenotypic variation in the SAM data is greatly affected by sampling time point, which an unsupervised method such as PCA does not distinguish.

### 3.3. 3D Root System Architecture Is Shaped by Genetics, Environment, and Development

**Figure 4:**
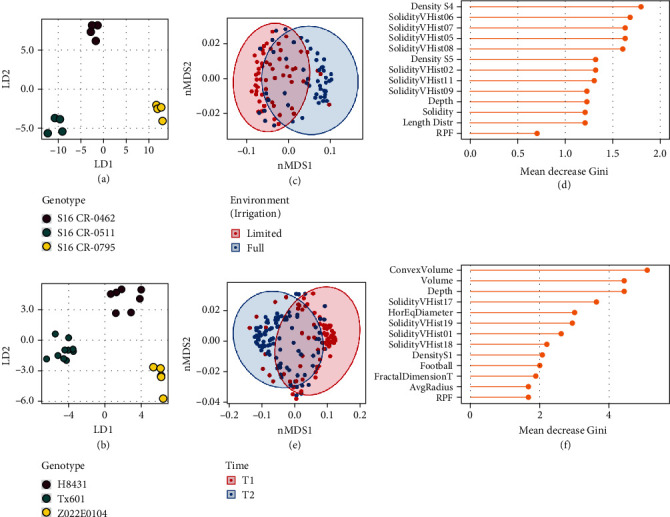
Classification based on genotype, environment, and time point using 3D root system architecture traits and RPF. Examples of highly distinguishable genotypes by PCA-LDA in the G2F 2017 data (a) and SAM 2018 data (b). Random forest classification of all samples based on environment in the G2F 2017 data (c) and importance of the 12 most influential traits plus RPF (d). Random forest classification of all samples based on time point in the SAM 2018 data (e) and importance of the 12 most influential traits plus RPF (f).

We next applied supervised multivariate classification methods to determine which traits were most closely associated with differences in genotype, environment, or time (Table S4). Because of the high number of genotypes (18 in the G2F set and 16 in the SAM set, after filtering for genotypes with the least missing data), in both experiments, the data was split into every possible combination of three genotypes, generating 816 different genotype combinations in the G2F set and 560 different genotype combinations in the SAM set. We performed PCA-LDA for genotype classification upon each three-genotype data subset, in each case using the minimum number of principal components to explain 90% of the variance (5-7 principal components with a median of 6 in G2F data; 9-13 principal components with a median of 11 in SAM data) as the inputs for LDA (Figures 4(a) and 4(b)). Across all genotype combination subsets, the average classification accuracy using leave-one-out cross-validation was 54.6% in the G2F and 67.2% in the SAM, both significantly higher than the 1/3 expected by random chance and therefore indicating that this approach is sensitive to distinguish between many of the genotypes, especially considering that numerous genotypes may in fact be phenotypically similar (Figure S1A).

We examined what traits were most important towards PCA-LDA classification across all genotype combinations (Figure S9-S10). In both the G2F and SAM populations, maximum root count, average root radius, and specific root length tended to be very important for genotype discrimination. Additionally, the median root count, number of root tips, elongation, and average edge length were important in genotypic classification among the G2F population, while several root solidity and density traits were important in genotypic classification among the SAM population. Although RPF was not among the top traits for genotypic classification, it was still well above average, ranking 12^th^ overall in the G2F set and 19^th^ overall in the SAM set. Interestingly, traits related to overall root size such as volume or surface area did not seem to be important factors overall in discriminating between genotypes, suggesting that these metrics, although intuitive and undoubtedly important in other contexts, are by themselves insufficient to distinguish between multiple and often subtly distinct genotypes, highlighting the need for the more comprehensive phenotyping described here to fully capture quantitative differences in root development.

To evaluate the overall effect of the environment (influential in the G2F experiment) and time (highly influential in the SAM experiment), we performed random forest classification to distinguish between the two possible levels of each variable upon root system architecture. For these classifications, we included all genotypes, which increased the sample size for each model. Using 10-fold cross-validation, the best model parameters resulted in a classification accuracy of 81.0%, indicating that while the environment had an effect which was detectable using classification techniques, the contrasting irrigation regimes were not so dramatic as to result in a shift in root system architecture obvious across every sample (Figure 4(c)). Nevertheless, changes in density and solidity distributions, as well as root crown depth, were the most distinguishing features, with RPF being less important (Figure 4(d)). Here, the importance of solidity distributions in the upper half of the root crown (including SolidityVHist 02-11) is consistent with ANOVA analysis (Figure S5A); in particular, the low broad-sense heritability of SolidityVHist 05-10, coupled with disproportionately high variance from environment and genotype-environment effects, indicates that these are more determined by environmental factors than by genetics in this experiment (Figure S3A-B). On the other hand, DensityS5 (a measure of relative compactness), which had a moderately high heritability in this experiment, is still important for distinguishing the effect of environment, suggesting that this trait is strongly affected by both genotype and environment.

For classifying roots based on time point in the SAM data, using 10-fold cross-validation, the best model parameters resulted in a random forest classification accuracy of 78.6% (Figure 4(e)), which was reasonable when considering that samples across both environmental conditions were included. Here, differences in convex hull volume, volume, depth, root crown width, and solidity distribution were the most distinguishing features, with RPF closely behind these and other important traits (Figure 4(f)). Furthermore, solidity distribution features at the very top and bottom of the root crown (SolidityVHist 01 and 17-19) appear to be relevant. These results are consistent with the increasing root crown size over time and with ANOVA results on time point effects upon these traits (Figure 2(b)).

Overall, multiple analytical approaches corroborate the conclusion that distinct sets of root traits are relevant depending on the germplasm, environment, and developmental time stage, reinforcing the relevance of high-dimensional root phenotyping. This also demonstrates not only the complexity of root system architectures, but its propensity to change under different contexts and genetic influences, and its ability to adapt to various conditions.

## 4. Discussion

We have shown that using X-ray computed tomography, changes in 3D root architecture between different treatments and conditions in the field can be measured in a biologically interpretable way, while also with high precision and detail. For example, soil moisture conditions affect the solidity of the root system, particularly in the midportion of the crown. In contrast, changes over time influence not only the overall size of the root crown but also solidity in the upper and lower portions of the crown. Interestingly, in both contexts, the depth (i.e., the length of the vertical axis) of the root crown also was a distinguishing feature. This is likely specific to root crowns excavated using the root pulling method, as under standard shovel excavation, the root depth would be more arbitrary unless carefully controlled for in the experimental design and execution. By using root pulling, however, the depth is a function of the root system and the soil conditions, as these determine where the root crown breaks and therefore encapsulates some information. Finally, in many instances, the 3D architectural measurements were easily sufficient to distinguish different maize genotypes, primarily using an entirely different set of traits including specific root length, median/maximum root count, and average root radius.

To date, 2D imaging has been by far the most popular form of quantifying root system architecture, whether in *Arabidopsis* grown on media plates, or root crowns excavated from the field. While relatively straightforward and convenient, 2D imaging does not represent true root system architecture in its natural form and therefore may omit important information. More recently, optical imaging platforms have been developed to perform 3D imaging of plants growing in gel media [37, 39, 50]. Here, we present a new approach to quantifying hundreds of field-excavated root crowns using X-ray CT, which is typically restricted to very small sample sizes or reconstituted soils from pot experiments [41]. Once the equipment and protocols are established, processing thousands of roots from a single field experiment and season is possible, making association mapping of 3D phenotypes feasible. Our results suggest that 3D imaging and the root system architectural traits derived from it have higher heritability (and therefore may be more informative) than methods using 2D imaging. Thus, future studies in quantitative root system architecture should increasingly utilize 3D phenotyping, either through XRT or the use of other imaging methods [62].

Still, the significant overhead associated with 3D imaging and analysis of roots will be a limitation to many researchers for the foreseeable future. We also addressed this by making explicit comparisons between high information content 3D phenotypes and root pulling force, which is accessible and can be scaled to high throughput levels. RPF measurements had several significant positive correlations with 3D architecture traits including volume, surface area, total root length, number of roots, and fraction dimension. Indeed, fractal dimension was a surprisingly powerful trait, not only being highly correlated with RPF but also having high heritability and contributing significantly to differentiating root system architecture over time. This is additional evidence that fractal dimension of root systems [53, 63–66] is a useful feature for quantifying maize root crowns under a variety of scenarios.

However, there remain sufficient sources of variance among features such that future improvements could strengthen the relationship between the above traits and RPF values. For example, we performed root pulling manually by hand, but a field robot or other form of mechanical assistance may result in more consistent RPF measurements [67]. Furthermore, while the fields were flooded just prior to root pulling to standardize the soil moisture conditions at the time of RPF sampling, local heterogeneity in soil texture or compactness may influence the measurements. This can be addressed in part by integrating larger studies whereby spatial effects can be modeled, which likewise would be facilitated with mechanization of the root pulling process. Finally, it should be noted that several traits not measurable by our XRT system could contribute to RPF, including the abundance of root hairs and variation in rhizosheath formation. Indeed, RPF heritability was notably higher later in development, consistent with a previous study showing high RPF heritability after maturity [19]. This observed difference might also suggest that RPF is controlled by different genetic factors over time, and therefore it may also be possible to select for early and later root phenotypes at least partially independently.

A better understanding of the exact nature and the potential interactions between the many root traits investigated here, such as exactly how fractal dimension and root volume together affect RPF, as well as the addition of more traits that could theoretically be calculated from 3D imaging (such as those relating to topology), may lead to additional insight into the relationship between root system architecture and RPF. As the interplay between these traits and above-ground performance such as yield and shoot biomass can be weak or inconsistent due to pleiotropy, linkage, and a gap in the understanding of root-shoot interactions, fully resolving how these traits interact would be beneficial for isolating desirable phenotypes and increasing selection efficiency within breeding applications. This would also be useful in multienvironment or stress-related studies, which typically require high sample sizes that the RPF method is well-suited for. Indeed, our study suggests that many more field-scale studies, utilizing wide-ranging conditions and germplasms, will be needed to fully characterize and understand quantitative root system architecture and genotype-environment interactions in diverse plant species such as maize.

## Data Availability

Raw extracted feature data is included as Data File S1. Images and image volumes are available upon request.

## Supplementary Materials

Supplementary Materials Figure S1: RPF distributions and example X-ray CT scan. Figure S2: correlation between 2D and 3D RSA traits. Figure S3-S4: heritability and variance component analysis of RSA traits. Figure S5: GxE in 3D RSA traits and regression between select traits vs. root mass. Figure S6-S7: significantly different 3D RSA traits between irrigation treatments and/or time point. Figure S8: PCA of 3D RSA traits. Figure S9-S10: trait importance across PCA-LDA. Table S1: genotypes used in X-ray CT. Table S2: 3D RSA trait descriptions. Table S3: correlation between RPF and 3D RSA traits. Table S4: accuracies of different classification methods for genotype and environment. Table S5: correlation between 3D RSA traits across time points. Data File S1: 2D and 3D RSA phenotype data.
