# Supplementary material for: Complementary Phenotyping of Maize Root System Architecture by Root Pulling Force and X-Ray Imaging
Source: Plant Phenomics. 2021 Nov 10;2021:9859254. doi: 10.34133/2021/9859254 (PMC8603028; doi:10.34133/2021/9859254)

Supplemental Figures S1-S9

Figure S1: (A) Distribution of RPF values across all genotypes from G2F 2017 population (top) and genotypes from this population selected for X-ray CT (bottom). Example root crown from genotype S16 CR-0511 in the G2F 2017 experiment showing the X-ray CT reconstructed 3D volume (B), point cloud (C), close-up of a region in the point cloud (D), skeleton (E), and an independent 2D image (F).

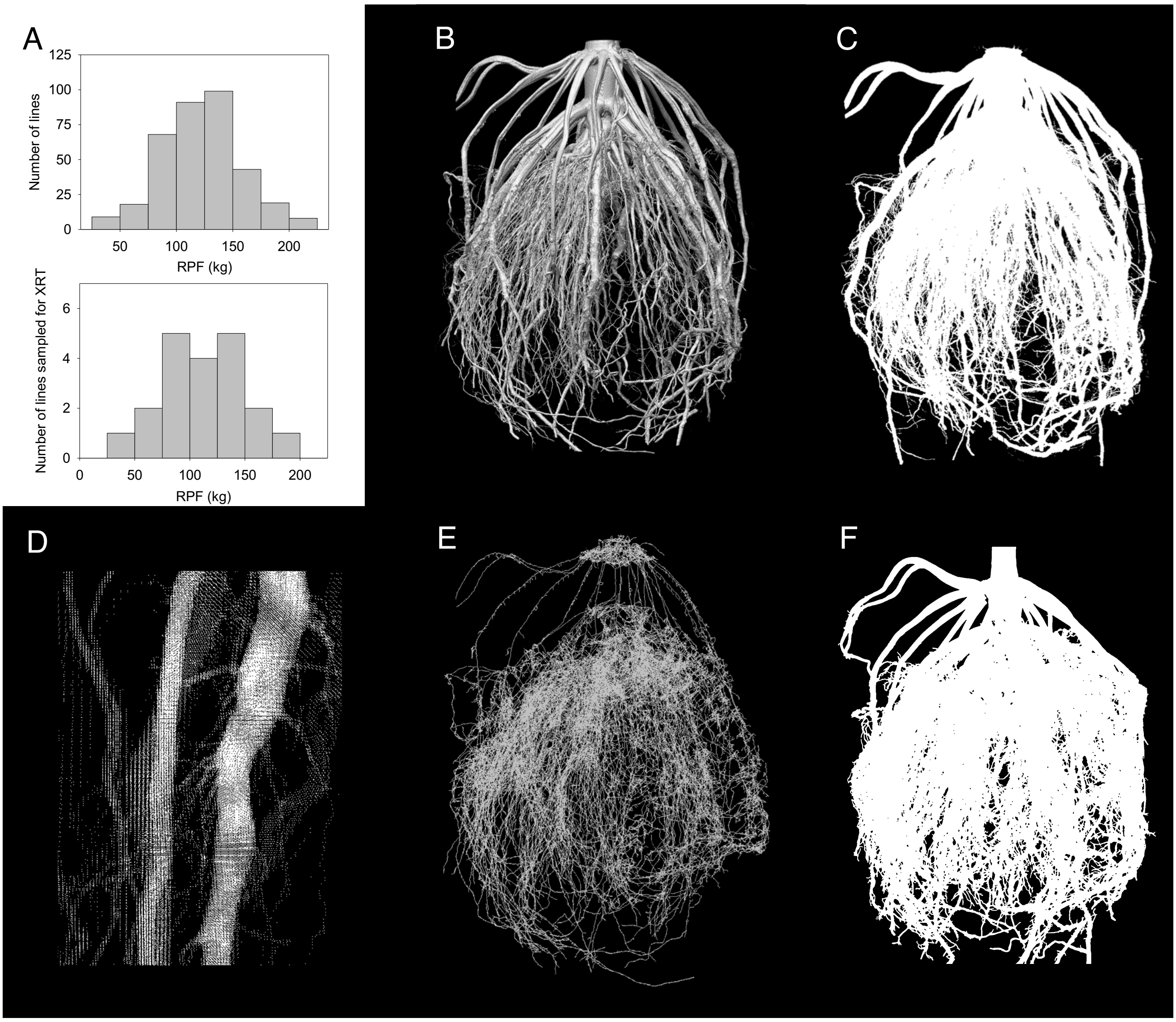

Figure S2: Heatmap of Pearson correlation between 2D and 3D traits from the G2F 2017 root crown samples. For brevity, only traits with a correlation coefficient ≥ 0.75 to at least one other trait were included, resulting in 32 2D traits and 54 3D traits kept. Traits were clustered based on complete linkage using Euclidean distances calculated between the correlation coefficients.

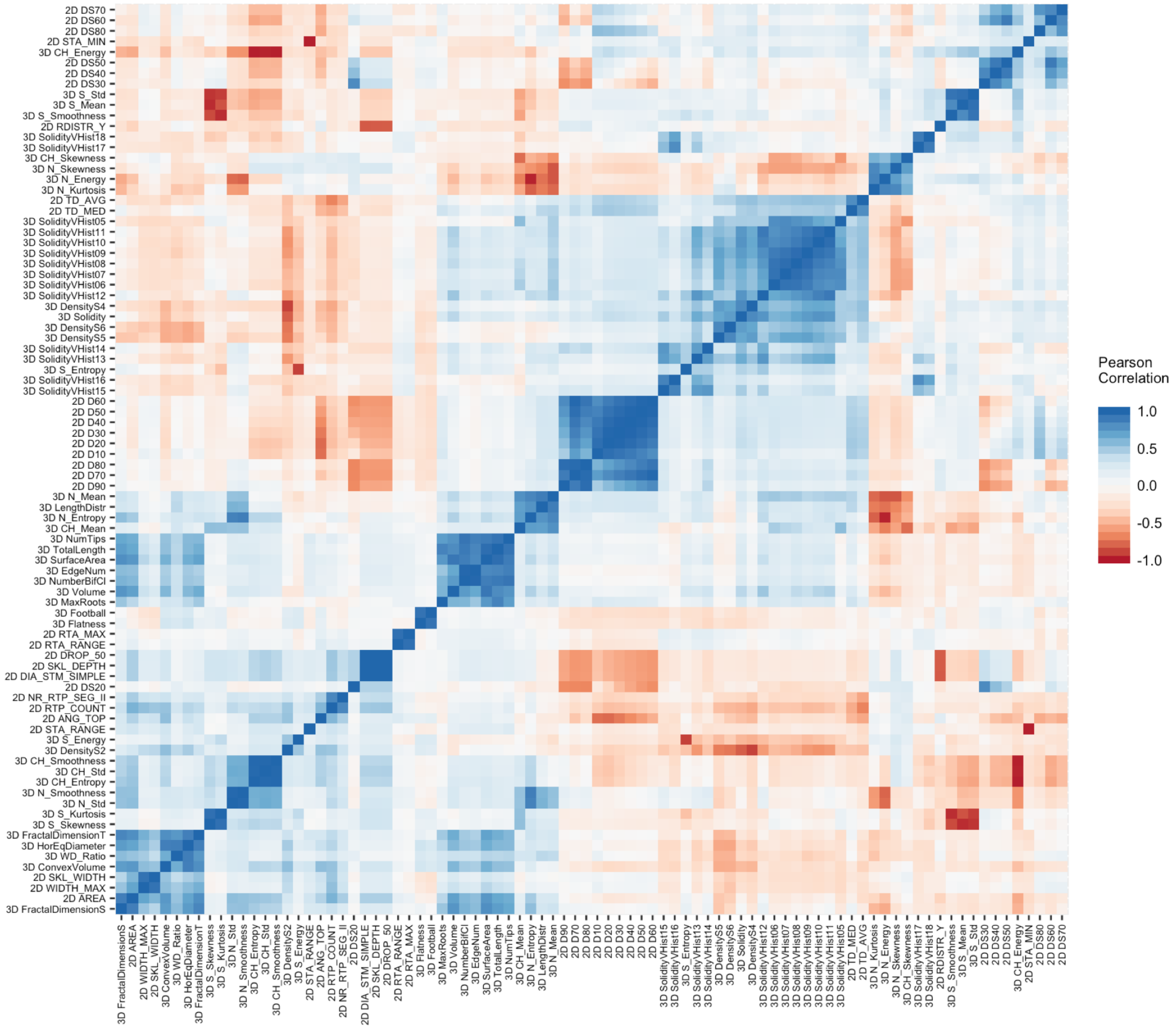

Figure S3: Broad-sense heritability (A) and variance component analysis (B) of RPF plus all 3D root traits from the G2M 2017 experiment. Broad-sense heritability (C) and variance component analysis (D) of 2D roots traits with heritability > 0 from the G2M 2017 experiment. Linear regression between RPF and 2D root area (E) or NR_RTP_SEG_II (F).

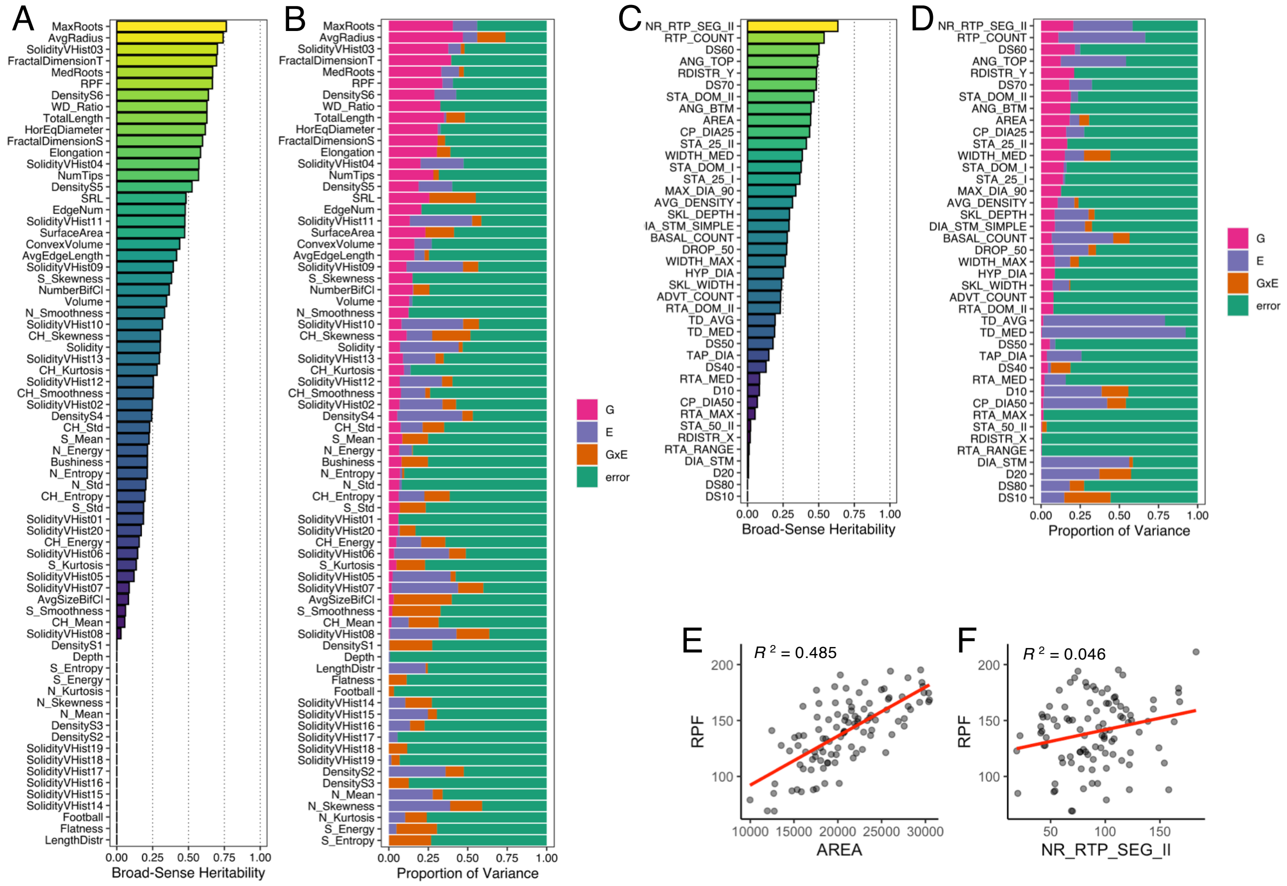

Figure S4: Broad-sense heritability of RPF and all 3D root traits from the SAM 2018 experiment at both time points (A). Variance components for each trait in time point 1 (B) and time point 2 (C). Traits in A-C are ordered by mean heritability across both time points. Scatterplot of mean heritability across both time points versus Spearman correlation between both time points, with each trait as a single point (D); correlation values for all traits are contained in Table S5.

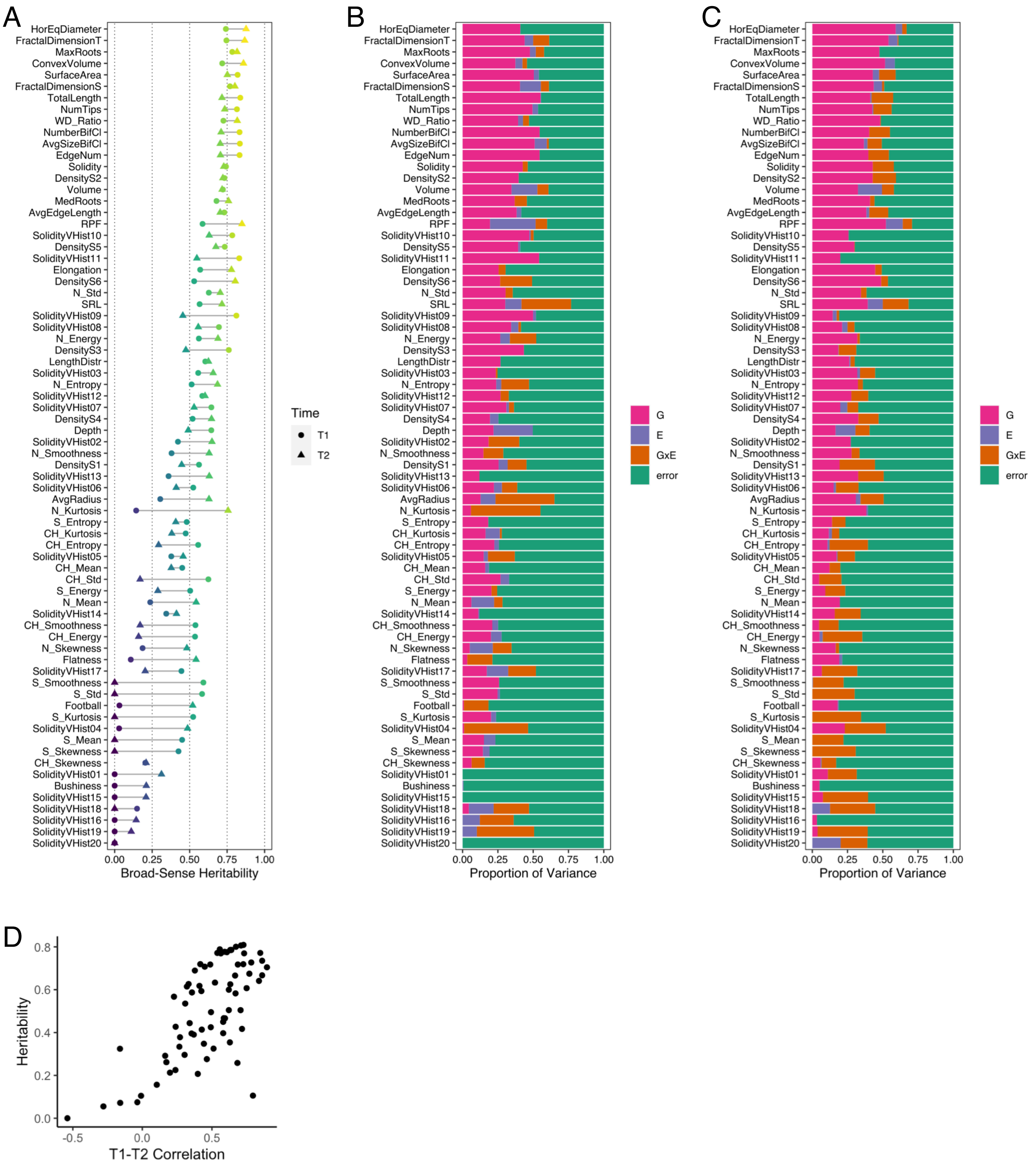

Figure S5: RPF and 3D distributional root system architecture traits are affected by genotype, environment, and developmental time point. There are consistent relationships between root biomass and RPF or 3D root traits across both time points (B-H), and a non-linear relationship between fractal dimension side/top and root biomass (I-J).

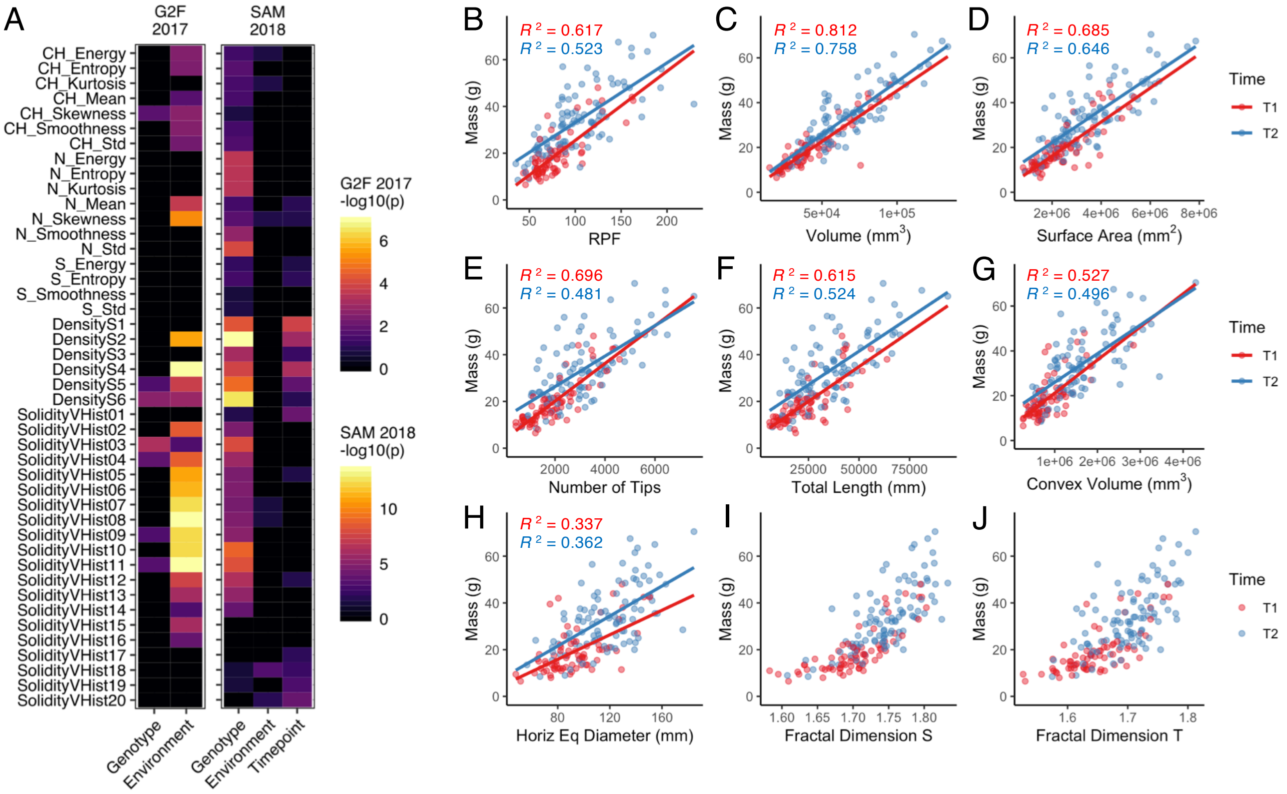

Figure S6: Boxplots of values for all traits with significant differences (from ANOVA, adjusted p-values) between limited vs full irrigation in the G2F 2017 experiment.

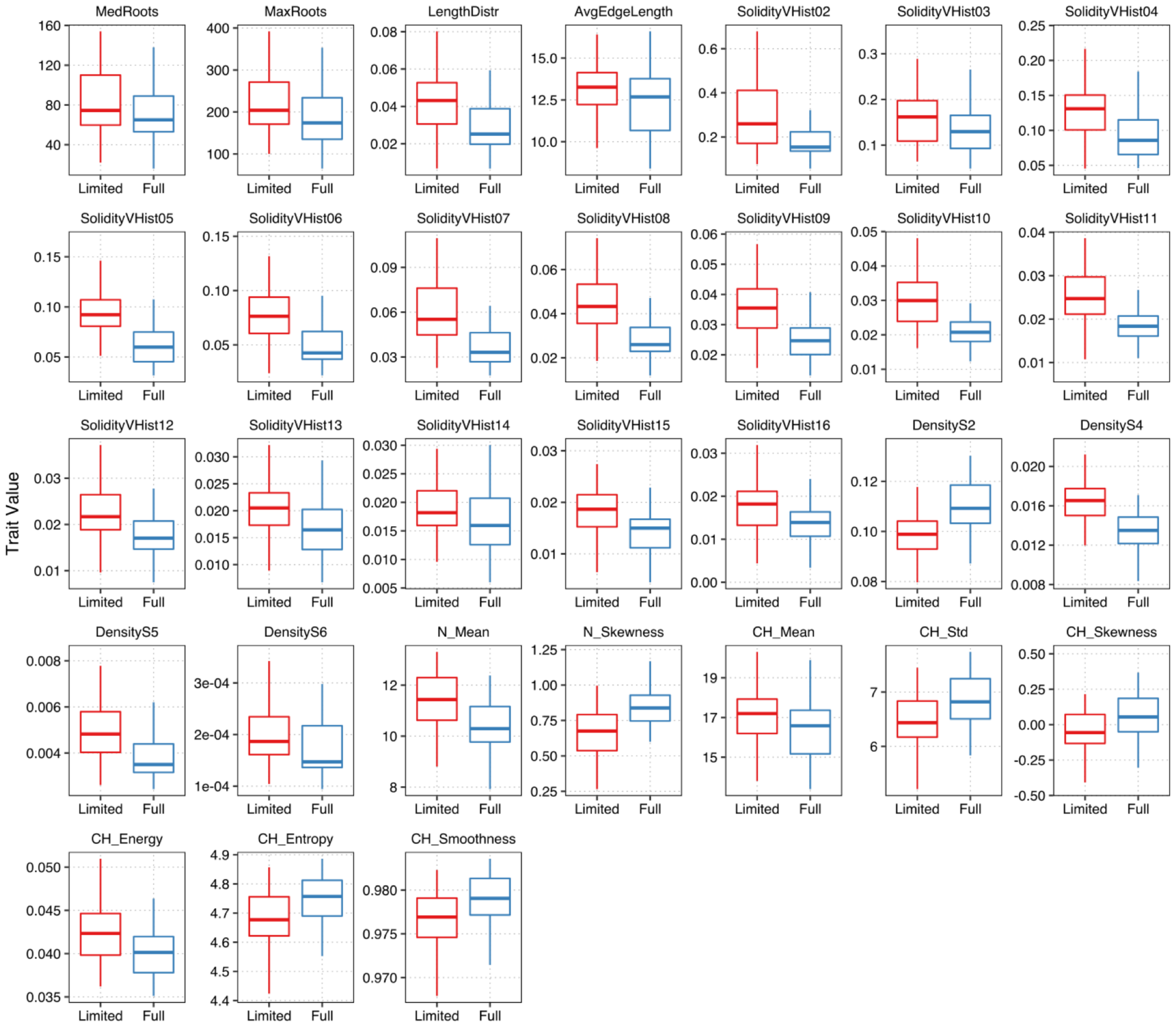

Figure S7: Boxplots of values for all traits with significant differences (from ANOVA, adjusted p-values) between the 1st and 2nd time point or limited vs full irrigation in the SAM 2018 experiment.

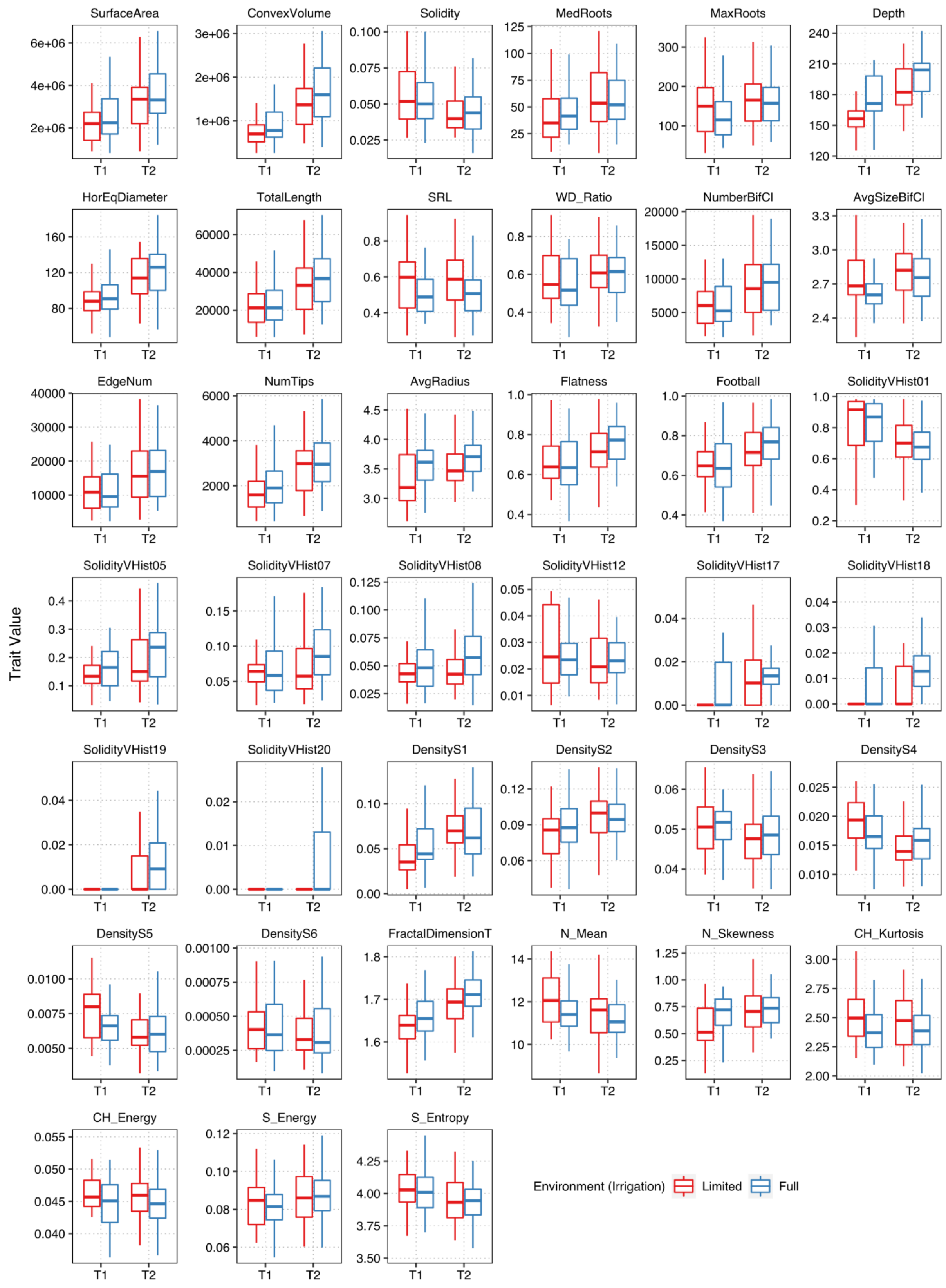

Figure S8: (A) Visualization of PC1 and PC2 from principal component analysis of the G2M 2017 3D root data, colored by RPF value (note: the +/- direction of PC values is arbitrary). Linear regression between G2M 2017 PC2 values and RPF (B), and boxplot of between limited vs full irrigation environments for PC1 (C) and PC2 (D). (E) Visualization of PC1 and PC2 from principal component analysis of the SAM 2018 3D root data, colored by RPF value. Linear regression between SAM 2018 PC1 values and RPF (F), and boxplot of between limited vs full irrigation environments and time point 1 vs time point 2 for PC1 (C) and PC2 (D). 3D root trait loading estimates for PC1 and PC2 from 500 re-samplings (75% of dataset per re-sample) from G2M 2017 (I) and SAM 2018 (J) data.

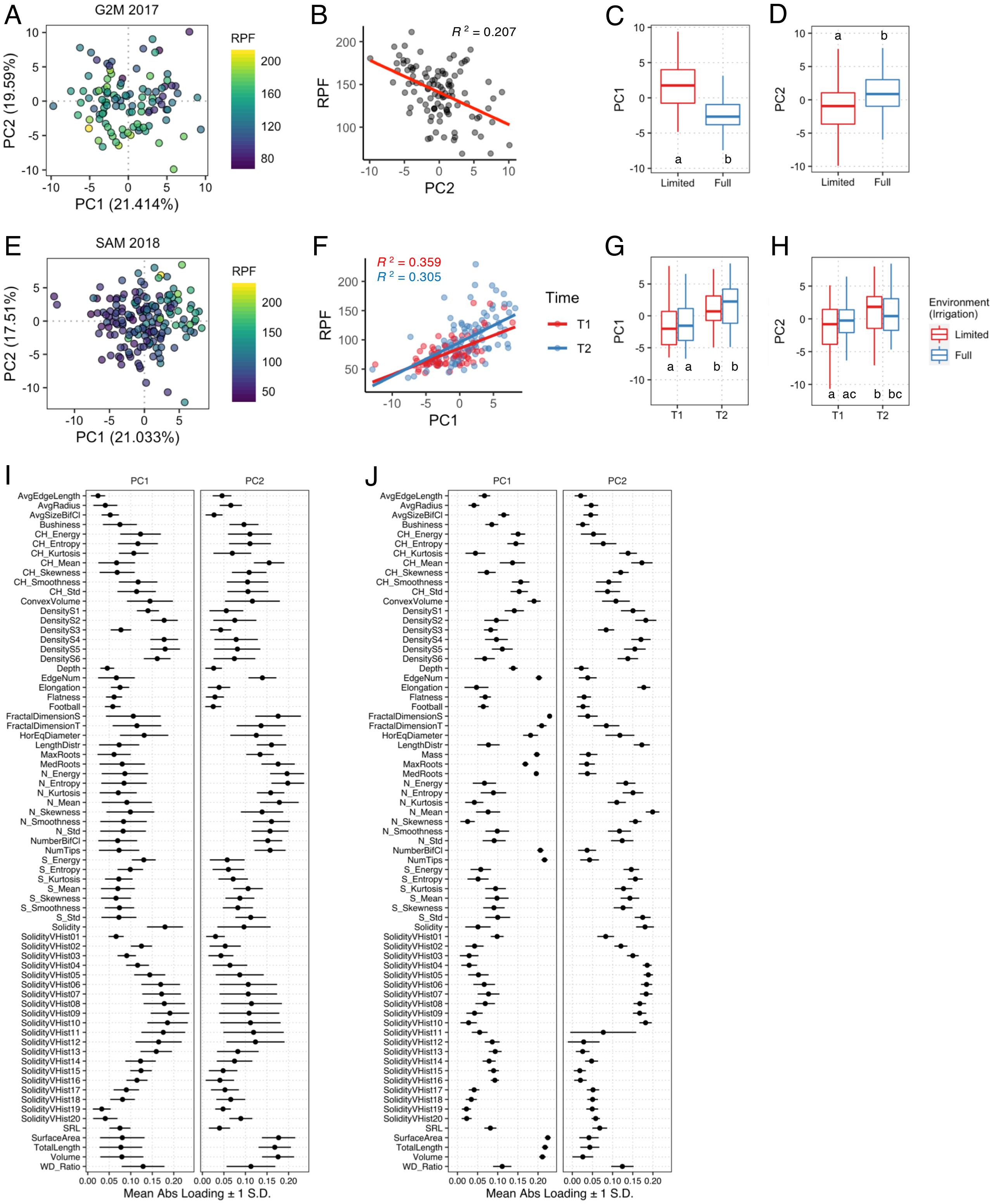

Figure S9: Histogram of rank for every root trait across all PCA-LDA permutations (i.e., all possible combinations of 3 genotypes), in terms of importance for classification in the G2F 2017 data. Leftmost bin corresponds to 1st rank (better), while rightmost bin corresponds to 72nd rank (worse). Dashed line indicates rank expected by random chance if all traits were equal in importance.

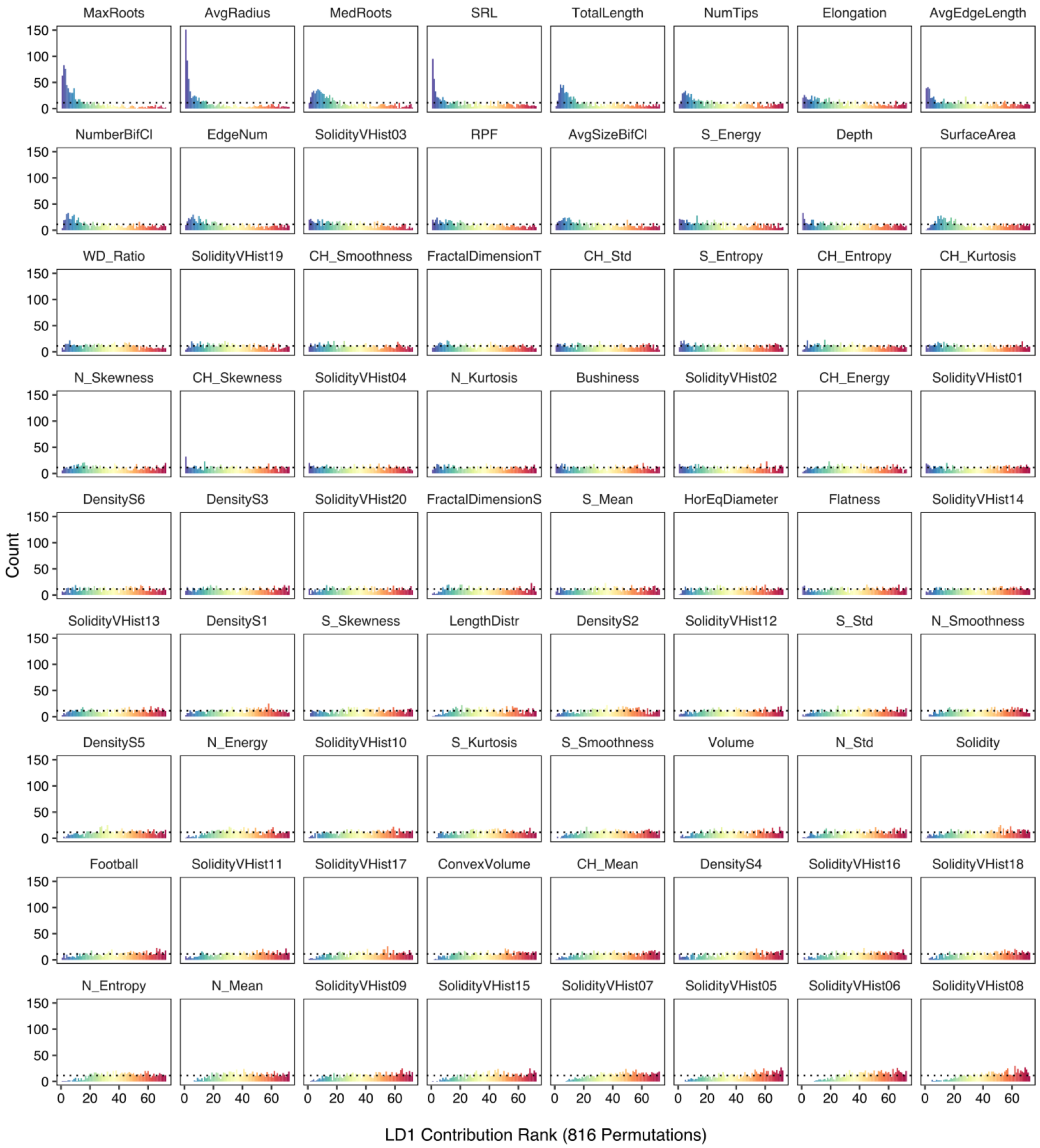

Figure S10: Histogram of rank for every root trait across all PCA-LDA permutations (i.e., all possible combinations of 3 genotypes), in terms of importance for classification in the SAM 2018 data. Leftmost bin corresponds to 1st rank (better), while rightmost bin corresponds to 72nd rank (worse). Dashed line indicates rank expected by random chance if all traits were equal in importance.

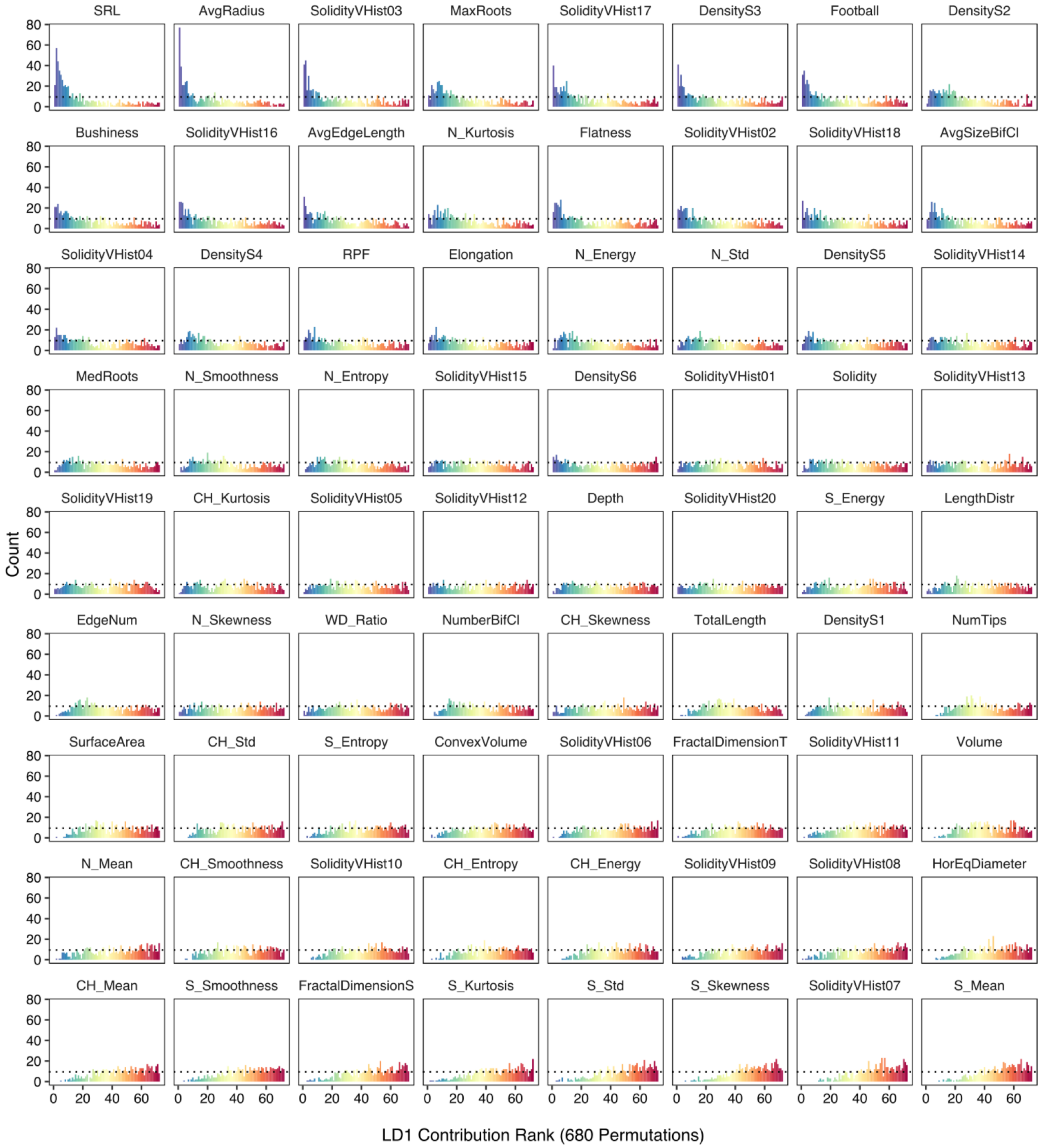

Supplement: Supplementary Materials — Figure S1: RPF distributions and example X-ray CT scan. Figure S2: correlation between 2D and 3D RSA traits. Figure S3-S4: heritability and variance component analysis of RSA traits. Figure S5: GxE in 3D RSA traits and regression between select traits vs. root mass. Figure S6-S7: significantly different 3D RSA traits between irrigation treatments and/or time point. Figure S8: PCA of 3D RSA traits. Figure S9-S10: trait importance across PCA-LDA. Table S1: genotypes used in X-ray CT. Table S2: 3D RSA trait descriptions. Table S3: correlation between RPF and 3D RSA traits. Table S4: accuracies of different classification methods for genotype and environment. Table S5: correlation between 3D RSA traits across time points. Data File S1: 2D and 3D RSA phenotype data. [file 9859254.f1.zip › Shao_PlantPhenomics_SupplementalFigures_Revised.pdf]
