# Supplementary material for: Complementary Phenotyping of Maize Root System Architecture by Root Pulling Force and X-Ray Imaging
Source: Plant Phenomics. 2021 Nov 10;2021:9859254. doi: 10.34133/2021/9859254 (PMC8603028; doi:10.34133/2021/9859254)
Supplement: Supplementary Materials — Figure S1: RPF distributions and example X-ray CT scan. Figure S2: correlation between 2D and 3D RSA traits. Figure S3-S4: heritability and variance component analysis of RSA traits. Figure S5: GxE in 3D RSA traits and regression between select traits vs. root mass. Figure S6-S7: significantly different 3D RSA traits between irrigation treatments and/or time point. Figure S8: PCA of 3D RSA traits. Figure S9-S10: trait importance across PCA-LDA. Table S1: genotypes used in X-ray CT. Table S2: 3D RSA trait descriptions. Table S3: correlation between RPF and 3D RSA traits. Table S4: accuracies of different classification methods for genotype and environment. Table S5: correlation between 3D RSA traits across time points. Data File S1: 2D and 3D RSA phenotype data. [file 9859254.f1.zip › Shao_SupplementalTables_RevisedFinal.docx]

**Tables S1-S5**

**Table S1:** Genotypes included in 3D imaging from the G2M and SAM experiments.

| **G2M 2017** | **SAM 2018** |
| --- | --- |
| 15SFG:2148 | 778 |
| 15SJWE:G2F:04026/04020 | A441-5 |
| 15SJWE:G2F:05026/05020 | CH9 |
| 15SJWE:G2F:09041/09020 | CM7 |
| 15SJWE:G2F:12036/12020 | CR14 |
| 15SJWE:G2F:13041/13020 | H8431 |
| 15SJWE:G2F:14051/14020 | LH146Ht |
| 15SJWE:G2F:15046/15020 | M162W |
| 15SJWE:G2F:15051/15020 | Mt42 |
| 15SJWE:G2F:16031/16020 | N59B |
| 16SJWE:G2F:25251/25265 | NC236 |
| CS15-YCB-17-X-POL-J4 | Os420 |
| S15 IB-0364 | P39 |
| S15 IB-0384 | Pa875 |
| S16 CR-0369 | T8 |
| S16 CR-0438 | Tx601 |
| S16 CR-0462 | Va17 |
| S16 CR-0477 | Va99 |
| S16 CR-0485 | Z022E0104 |
| S16 CR-0511 | ZS01250 |
| S16 CR-0519 |  |
| S16 CR-0569 |  |
| S16 CR-0580 |  |
| S16 CR-0621 |  |
| S16 CR-0682 |  |
| S16 CR-0722 |  |
| S16 CR-0795 |  |
| S16 IA-0687 |  |
| WISN15/30649 |  |
| WISN15/30853 |  |

**Table S2:** 3D traits descriptions; see Materials and Methods for the link to our repository and citations to literature the traits are derived from, where applicable. Data representations denote whether the computation was performed on the Point Cloud Data= PCD, the skeleton, or both.

| **3D Trait** | **Description** | **Data Representation(s)** |
| --- | --- | --- |
| SurfaceArea | Sum of exposed voxel faces on the surface of the root model. | PCD |
| Volume | The sum of each voxel in the root model; a typical proxy for “biomass” in digital phenotyping. | PCD |
| ConvexVolume | The volume of the convex hull that encompasses the root model. | PCD |
| Solidity | The volume divided by the convex volume, a measure of the thoroughness of root exploration. | PCD |
| MedRoots | Median number of roots among all horizontal slices. | PCD |
| MaxRoots | 84^th^ percentile value of the number of roots among all horizontal slices. | PCD |
| Bushiness | The ratio of the maximum to the median number of roots. | PCD |
| Depth | The number voxels of the vertical axis of the root model, a measure of the depth of the deepest root. | PCD |
| HorEqDiameter | Maximum root model width among all horizontal slices. | PCD |
| TotalLength | Root length as approximated by the number of voxels in the skeleton. | Skeleton |
| SRL | Specific root length; the total length divided by the volume, similar to the traditional measure of total length divided by the biomass. | Skeleton & PCD |
| LengthDistr | The ratio of root length in the upper 1⁄3 of the root model to the root length in the lower 2⁄3 of the model. | Skeleton & PCD |
| WD_Ratio | Width-to-depth ratio; the maximum root model width divided by the depth. | PCD |
| NumberBifCl | Estimated number of branching points in the skeleton. | Skeleton |
| AvgSizeBifCl | Estimated number of branches at each branching point in the skeleton. | Skeleton |
| EdgeNum | Number of skeleton segments between estimated branching points | Skeleton |
| AvgEdgeLength | Average length of skeleton segments between estimated branching points, a measure of branching density of the root system. | Skeleton |
| NumberTips | Number of root tips in the root model. | Skeleton |
| AvgRadius | The average radius of all roots in the model, as estimated by the distance of each voxel in the skeleton from the surface of the root model. | Skeleton & PCD |
| Elongation | PCA on 3D point cloud, taking the ratio between PC2 variance and PC1 variance; measures how elongated the root is. | PCD |
| Flatness | PCA on 3D point cloud, taking the ratio between PC3 variance and PC2 variance; measures how flat the root is. | PCD |
| Football | PCA on (x, y) of 3D point cloud, taking the ratio between PC2 variance and PC1 variance. | PCD |
| SolidityVHist 01-20 | The solidity at each slice is computed, then spline interpolated to the n^t​h^​ cm (1-20) below the top. | PCD |
| DensityS 1-6 | The frequency of voxels with different 6 overlap ratios from side view. S6 represents the largest overlap ratio. Higher numbers in greater overlap ratio means a denser root. | PCD |
| FractalDimensionS | Fractal dimension is estimated from the projected side-view image using the box-counting method. It is a measure of how complicated a root shape is using self-similarity. | PCD |
| FractalDimensionT | Fractal dimension estimated from the projected top-view image using the box-counting method. It is a measure of how complicated a root shape is using self-similarity. | PCD |
| N/CH/S Mean | Mean estimated from the distribution of biomass/volume (N), convex hull (CH), or solidity (S) along the z-axis. | PCD |
| N/CH/S Std | Standard deviation estimated from the distribution of biomass/volume (N), convex hull (CH), or solidity (S) along the z-axis. | PCD |
| N/CH/S Skewness | Skewness, or inequality, estimated from the distribution of biomass/volume (N), convex hull (CH), or solidity (S) along the z-axis. Negative value indicates that a large number of the values are lower than the mean (left-tailed); positive value indicates that a larger number of the values are higher than the mean (right-tailed). | PCD |
| N/CH/S Kurtosis | Kurtosis, or peakiness, estimated from the distribution of biomass/volume (N), convex hull (CH), or solidity (S) along the z-axis. High value indicates that the peak of the distribution around the mean is sharp and long-tailed; low value indicates that the peak around the mean is round and short-tailed. | PCD |
| N/CH/S Energy | Energy, or uniformity, estimated from the distribution of biomass/volume (N), convex hull (CH), or solidity (S) along the z-axis. A high value indicates that the distribution has a small number of different levels. | PCD |
| N/CH/S Entropy | Entropy, the inverse of energy, estimated from the distribution of biomass/volume (N), convex hull (CH), or solidity (S) along the z-axis. A high value indicates that the distribution has a higher number of different levels. | PCD |
| N/CH/S Smoothness | Smoothness estimated from the distribution of biomass/volume (N), convex hull (CH), or solidity (S) along the z-axis. Defined as $1-\frac{1}{1+{(stddev)}^{2}}$ | PCD |

**Table S3:** Spearman correlation between RPF and each 3D trait using every root sample as an observation. Adjusted p-values were calculated using the Benjamini-Hochberg method.

| **G2M 2017** | | | **SAM 2018** | | |
| --- | --- | --- | --- | --- | --- |
| **3D Trait** | **Correlation to RPF** | **Adj. p-value** | **3D Trait** | **Correlation to RPF** | **Adj. p-value** |
| FractalDimensionS | 0.64367077 | 9.25E-12 | Volume | 0.77528044 | 1.06E-33 |
| SurfaceArea | 0.61848397 | 7.31E-11 | FractalDimensionS | 0.749712 | 1.44E-30 |
| FractalDimensionT | 0.56966543 | 5.37E-09 | ConvexVolume | 0.74479439 | 3.95E-30 |
| Volume | 0.56082232 | 8.71E-09 | SurfaceArea | 0.70736033 | 5.26E-26 |
| TotalLength | 0.54212012 | 3.31E-08 | FractalDimensionT | 0.6883679 | 3.41E-24 |
| MedRoots | 0.53117402 | 6.58E-08 | HorEqDiameter | 0.66922067 | 1.72E-22 |
| ConvexVolume | 0.51922444 | 1.41E-07 | Depth | 0.64920791 | 7.84E-21 |
| HorEqDiameter | 0.50256974 | 4.16E-07 | TotalLength | 0.6260746 | 4.75E-19 |
| NumTips | 0.50041339 | 4.30E-07 | NumTips | 0.61795041 | 1.72E-18 |
| NumberBifCl | 0.43870857 | 2.03E-05 | NumberBifCl | 0.56105043 | 1.03E-14 |
| WD_Ratio | 0.41691289 | 5.73E-05 | SolidityVHist19 | 0.54799953 | 5.59E-14 |
| EdgeNum | 0.40390322 | 0.00010545 | EdgeNum | 0.54337232 | 9.49E-14 |
| MaxRoots | 0.38046053 | 0.00031647 | MedRoots | 0.46546205 | 7.71E-10 |
| N_Std | 0.36549113 | 0.00052685 | SolidityVHist18 | 0.45245647 | 2.64E-09 |
| N_Smoothness | 0.36549113 | 0.00052685 | DensityS1 | 0.44178551 | 6.91E-09 |
| CH_Entropy | 0.33243572 | 0.00209331 | SolidityVHist20 | 0.37134304 | 2.39E-06 |
| CH_Std | 0.31746048 | 0.00343406 | WD_Ratio | 0.33520294 | 2.77E-05 |
| CH_Smoothness | 0.31746048 | 0.00343406 | MaxRoots | 0.32494819 | 5.14E-05 |
| Elongation | 0.30162996 | 0.00597165 | SolidityVHist17 | 0.30719348 | 0.00014782 |
| N_Entropy | 0.29730689 | 0.00639042 | DensityS2 | 0.29980008 | 0.000219 |
| DensityS3 | 0.26218325 | 0.01748469 | CH_Std | 0.28322805 | 0.00049489 |
| S_Entropy | 0.25018855 | 0.02460862 | CH_Smoothness | 0.28322805 | 0.00049489 |
| SolidityVHist18 | 0.22670713 | 0.04446167 | SolidityVHist07 | 0.27461444 | 0.00075956 |
| AvgEdgeLength | 0.2223426 | 0.04868217 | CH_Entropy | 0.27089083 | 0.0008911 |
| SolidityVHist17 | 0.18297364 | 0.11532375 | SolidityVHist06 | 0.2490196 | 0.00250245 |
| SolidityVHist12 | 0.18170367 | 0.11575301 | SolidityVHist08 | 0.24575559 | 0.00282951 |
| SolidityVHist14 | 0.17664973 | 0.12680958 | Football | 0.24146799 | 0.00335381 |
| N_Mean | 0.15111563 | 0.19541674 | S_Energy | 0.23989478 | 0.00349689 |
| SolidityVHist16 | 0.14876748 | 0.20045463 | Flatness | 0.23852558 | 0.00355067 |
| LengthDistr | 0.14141796 | 0.22671434 | AvgRadius | 0.23362971 | 0.00426487 |
| DensityS2 | 0.14035461 | 0.22671434 | CH_Mean | 0.21543874 | 0.00893575 |
| SRL | 0.12237598 | 0.30954307 | AvgSizeBifCl | 0.18869802 | 0.02386921 |
| S_Std | 0.11599717 | 0.33223615 | N_Std | 0.17651548 | 0.03536019 |
| S_Smoothness | 0.11599717 | 0.33223615 | N_Smoothness | 0.17651548 | 0.03536019 |
| SolidityVHist15 | 0.11139937 | 0.3521347 | SolidityVHist05 | 0.17136987 | 0.04130921 |
| SolidityVHist20 | 0.10881279 | 0.35625924 | Elongation | 0.14747544 | 0.08372401 |
| SolidityVHist13 | 0.10841366 | 0.35625924 | SolidityVHist09 | 0.14662223 | 0.08409402 |
| S_Mean | 0.09929063 | 0.39313407 | N_Skewness | 0.13837582 | 0.09875453 |
| CH_Skewness | 0.08317502 | 0.48005671 | S_Kurtosis | 0.1221506 | 0.14539334 |
| AvgSizeBifCl | 0.06146119 | 0.61271982 | LengthDistr | 0.1069311 | 0.20416743 |
| SolidityVHist19 | 0.0464704 | 0.68948129 | N_Entropy | 0.09620175 | 0.24931259 |
| Solidity | 0.04604339 | 0.68948129 | S_Skewness | 0.09149535 | 0.27233255 |
| SolidityVHist11 | 0.01985294 | 0.85965405 | SolidityVHist04 | 0.08171773 | 0.32456168 |
| Depth | 0.0150373 | 0.87898932 | SolidityVHist10 | 0.07967893 | 0.33306625 |
| Football | -0.0189873 | 0.85965405 | N_Kurtosis | 0.03698406 | 0.66872693 |
| Flatness | -0.0305984 | 0.79004999 | SolidityVHist11 | 0.01752003 | 0.85626075 |
| SolidityVHist10 | -0.0363677 | 0.75518085 | SolidityVHist16 | 0.01251722 | 0.88598271 |
| CH_Mean | -0.0475486 | 0.68948129 | N_Mean | -0.0091104 | 0.90585945 |
| DensityS1 | -0.0551425 | 0.64955312 | SRL | -0.0122645 | 0.88598271 |
| S_Energy | -0.0612124 | 0.61271982 | AvgEdgeLength | -0.0563797 | 0.499047 |
| N_Skewness | -0.0775509 | 0.51081248 | N_Energy | -0.0583801 | 0.48954141 |
| DensityS4 | -0.092184 | 0.42800555 | SolidityVHist12 | -0.0824413 | 0.32456168 |
| SolidityVHist09 | -0.098876 | 0.39313407 | SolidityVHist03 | -0.101321 | 0.22538969 |
| SolidityVHist08 | -0.0989693 | 0.39313407 | SolidityVHist13 | -0.1058246 | 0.20607144 |
| S_Skewness | -0.1634628 | 0.15439143 | SolidityVHist02 | -0.1134866 | 0.17677503 |
| S_Kurtosis | -0.1704865 | 0.13550835 | SolidityVHist14 | -0.121763 | 0.14539334 |
| DensityS6 | -0.1737469 | 0.12898261 | S_Std | -0.1371182 | 0.09875453 |
| SolidityVHist07 | -0.1740994 | 0.12898261 | S_Smoothness | -0.1371182 | 0.09875453 |
| SolidityVHist05 | -0.1860423 | 0.11016656 | SolidityVHist15 | -0.1392362 | 0.09845089 |
| SolidityVHist01 | -0.1869235 | 0.11016656 | S_Mean | -0.1442558 | 0.08662018 |
| Bushiness | -0.2138888 | 0.05773223 | CH_Kurtosis | -0.1455866 | 0.08496011 |
| SolidityVHist04 | -0.2171124 | 0.05450343 | CH_Skewness | -0.151475 | 0.07567575 |
| SolidityVHist06 | -0.2285888 | 0.04352637 | DensityS3 | -0.1658295 | 0.04871094 |
| AvgRadius | -0.2401429 | 0.03220121 | DensityS6 | -0.1901895 | 0.0231517 |
| N_Kurtosis | -0.2671594 | 0.01543081 | Solidity | -0.1902681 | 0.0231517 |
| N_Energy | -0.274349 | 0.01262625 | S_Entropy | -0.2238772 | 0.00639915 |
| DensityS5 | -0.2765261 | 0.01220194 | SolidityVHist01 | -0.2382604 | 0.00355067 |
| SolidityVHist03 | -0.2864474 | 0.00902986 | Bushiness | -0.2658881 | 0.00111542 |
| SolidityVHist02 | -0.2994943 | 0.00616708 | CH_Energy | -0.2873189 | 0.00042849 |
| CH_Energy | -0.3765683 | 0.00035587 | DensityS4 | -0.358491 | 5.82E-06 |
| CH_Kurtosis | -0.4309385 | 2.87E-05 | DensityS5 | -0.4100719 | 1.13E-07 |

**Table S4:** Classification accuracy summary from PCA-LDA (LOOCV) and random forest (10-fold CV). For PCA-LDA, sample size and number of groups (genotypes) per permutation is indicated. For random forest, number of groups (environments or time points) and sample size of the total model is indicated.

| **Data** | **Grouping** | **# Groups (Per Permutation)** | **Method** | **Sample Size  (Per Permutation)** | **Accuracy** |
| --- | --- | --- | --- | --- | --- |
| G2M 2017 | Genotype | 3 | PCA-LDA | 12 | 54.6% |
| G2M 2017 | Environment | 2 | Random Forest | 105 | 81.0% |
| SAM 2018 | Genotype | 3 | PCA-LDA | 27.75 (mean) | 67.2% |
| SAM 2018 | Environment | 2 | Random Forest | 171 | 66.2% |
| SAM 2018 | Time Point | 2 | Random Forest | 171 | 78.6% |

**Table S5:** Spearman correlation between T1 and T2 for each 3D trait using the average value for each genotype within time point as an observation.

| **3D Trait** | **T1-T2 Correlation** |
| --- | --- |
| DensityS5 | 0.894736842105263 |
| DensityS6 | 0.86015037593985 |
| Solidity | 0.86015037593985 |
| WD_Ratio | 0.846616541353383 |
| SRL | 0.837593984962406 |
| Bushiness | 0.793984962406015 |
| DensityS2 | 0.781954887218045 |
| Elongation | 0.768421052631579 |
| SolidityVHist03 | 0.748872180451128 |
| AvgSizeBifCl | 0.730827067669173 |
| HorEqDiameter | 0.726315789473684 |
| MedRoots | 0.724812030075188 |
| SolidityVHist05 | 0.715789473684211 |
| FractalDimensionT | 0.708270676691729 |
| DensityS1 | 0.705263157894737 |
| AvgEdgeLength | 0.685714285714286 |
| SolidityVHist04 | 0.682706766917293 |
| MaxRoots | 0.67218045112782 |
| DensityS4 | 0.669172932330827 |
| N_Std | 0.666165413533835 |
| SurfaceArea | 0.639097744360902 |
| N_Energy | 0.631578947368421 |
| FractalDimensionS | 0.631578947368421 |
| CH_Smoothness | 0.628571428571429 |
| N_Smoothness | 0.621052631578947 |
| N_Entropy | 0.621052631578947 |
| NumTips | 0.607518796992481 |
| AvgRadius | 0.593984962406015 |
| TotalLength | 0.590977443609023 |
| SolidityVHist06 | 0.584962406015038 |
| CH_Std | 0.580451127819549 |
| N_Kurtosis | 0.580451127819549 |
| EdgeNum | 0.56390977443609 |
| ConvexVolume | 0.556390977443609 |
| NumberBifCl | 0.538345864661654 |
| SolidityVHist09 | 0.521804511278195 |
| Flatness | 0.511278195488722 |
| SolidityVHist13 | 0.493233082706767 |
| CH_Entropy | 0.491729323308271 |
| RPF | 0.487719298245614 |
| Football | 0.463157894736842 |
| SolidityVHist10 | 0.44812030075188 |
| CH_Energy | 0.442105263157895 |
| CH_Mean | 0.427067669172932 |
| SolidityVHist12 | 0.42406015037594 |
| Volume | 0.416541353383459 |
| DensityS3 | 0.410526315789474 |
| CH_Skewness | 0.398496240601504 |
| SolidityVHist11 | 0.377443609022556 |
| Mass | 0.371929824561404 |
| N_Mean | 0.369924812030075 |
| SolidityVHist07 | 0.356390977443609 |
| S_Energy | 0.353383458646617 |
| S_Entropy | 0.33984962406015 |
| SolidityVHist08 | 0.332330827067669 |
| LengthDistr | 0.320300751879699 |
| SolidityVHist02 | 0.308270676691729 |
| S_Smoothness | 0.303759398496241 |
| SolidityVHist14 | 0.270676691729323 |
| N_Skewness | 0.266165413533835 |
| CH_Kurtosis | 0.239097744360902 |
| S_Mean | 0.237593984962406 |
| Depth | 0.227067669172932 |
| S_Skewness | 0.198496240601504 |
| S_Kurtosis | 0.172932330827068 |
| S_Std | 0.16390977443609 |
| SolidityVHist01 | 0.103759398496241 |
| SolidityVHist15 | -0.00902255639097744 |
| SolidityVHist18 | -0.0360902255639098 |
| SolidityVHist16 | -0.159398496240601 |
| SolidityVHist17 | -0.160902255639098 |
| SolidityVHist19 | -0.279699248120301 |
| SolidityVHist20 | -0.539157237939949 |
